# The contributing external load factors to internal load during small-sided games in professional rugby union players

**DOI:** 10.3389/fspor.2023.1092186

**Published:** 2023-02-15

**Authors:** Marco Zanin, Adelchi Azzalini, Jayamini Ranaweera, Dan Weaving, Joshua Darrall-Jones, Gregory Roe

**Affiliations:** ^1^ Carnegie Applied Rugby Research Centre, Leeds Beckett University, Leeds, United Kingdom; ^2^ Performance Department, Bath Rugby Football Club, Bath, United Kingdom; ^3^ Dipartimento di Scienze Statistiche, Università Degli Studi di Padova, Padua, Italy; ^4^ Performance Department, Leeds Rhinos Rugby League Club, Leeds, United Kingdom

**Keywords:** coaching, practice design, constraints, training stress, likelihood inference, outliers

## Abstract

**Introduction:**

This study aimed to investigate which external load variables were associated with internal load during three small-sided games (SSG) in professional rugby union players.

**Methods:**

Forty professional rugby union players (22 forwards, 18 backs) competing in the English Gallagher Premiership were recruited. Three different SSGs were designed: one for backs, one for forwards, and one for both backs and forwards. General linear mixed-effects models were implemented with internal load as dependent variable quantified using Stagno's training impulse, and external load as independent variables quantified using total distance, high-speed (>61% top speed) running distance, average acceleration-deceleration, PlayerLoad™, PlayerLoad™ slow (<2 m·s^−1^), number of get-ups, number of first-man-to-ruck.

**Results:**

Internal load was associated with different external load variables dependent on SSG design. When backs and forwards were included in the same SSG, internal load differed between positional groups (MLE = −121.94, SE = 29.03, *t* = −4.20).

**Discussion:**

Based on the SSGs investigated, practitioners should manipulate different constraints to elicit a certain internal load in their players based on the specific SSG design. Furthermore, the potential effect of playing position on internal load should be taken into account in the process of SSG design when both backs and forwards are included.

## Introduction

Rugby union players need to develop multiple qualities (e.g., technical skills, cardiovascular capacity) to succeed in their sport (1). Whilst each component could be trained in isolation, small-sided game (SSG) training is a commonly used training method to enhance technical skills, tactical understanding, and physical qualities concurrently (2). Small-sided games resemble aspects of official games as they involve two teams competing against each other, and hence are characterised by high unpredictability and decision-making activities (2).

Practitioners generally design training drills, such as tactical, or SSG training, using external load variables and by considering the whole team as a collective (3). External load has been identified as the activities prescribed to and completed by athletes, and can be quantified using technologies such as global navigation satellite systems (GNSS) or tri-axial accelerometer and metrics such as total distance covered and PlayerLoad™ (4). Conversely, internal load has been identified as the psycho-physiological response of the individual to the external load, and can be quantified using objective, such as heart rate monitors, and subjective measures, such as rating of perceived exertion (RPE) (4). In the process of SSG design, sport coaches and sport scientists may need to understand which external load variables—potentially dictated by the constraints of the SSG (e.g., rules, number of players)—would result in a certain internal load (3). This information would allow practitioners to manipulate constraints to find a balance in the development of technical skills, tactical understanding, and physical qualities.

Previous research found that internal load (i.e., session-RPE) was affected by external load, playing position, and cardiovascular capacity during the combination of technical, tactical and SSG training, throughout a pre-season in professional Australian football players (3). Additionally, during association football training sessions, internal load (i.e., session-RPE) was influenced by total distance covered, total duration of training, number of sprints (>25 km·h^−1^), high-speed (>14.4 km·h^−1^) running distance covered, and number of impacts and accelerations (>3 m·s^−2^) (5, 6). However, in these studies, technical, tactical, and SSG training were grouped and investigated together. Consequently, the findings may not be specific enough to support practitioners in the process of SSG design.

Different training methods may elicit different external and internal loads which may also result in different adaptations (7). For instance, SSG training resulted in higher external load (PlayerLoad™·min^−1^) in comparison with technical and tactical training in professional Australian football players (7). In addition, the strength of the correlation between external (e.g., BodyLoad™) and internal load (i.e., session RPE) changed based on the training method investigated (e.g., wrestling, SSG training) (8). Consequently, knowledge of the external load variables contributing to internal load for specific training methods (e.g., SSG training) would better support practitioners in the process of practice design.

Due to the widespread use of SSG training and the limited research available in rugby union on the topic (9), this study aimed to investigate which external load variables were associated with internal load during multiple SSGs in professional rugby union players. It was hypothesised that different external load variables would offer a different contribution to internal load, based on the specific design of the SSG.

## Methods

### Subjects

Forty professional rugby union players (Table 1) from the same rugby union club competing in the English Gallagher Premiership were involved in this study. The sample from this study was based on the availability of players at the professional rugby union club throughout pre-season 2019/2020, hence it was derived from convenience sampling. Informed and written consent was received by all the participants before the start of data collection. The protocol of the study followed the guidelines of the Declaration of Helsinki and received ethical approval from Leeds Beckett University Ethics Committee (ethics ID: 82039).

**Table 1:** Individual characteristics for the participants of the study.

Individual characteristics			
Variable	Forwards	Backs	Forwards and backs
Sample size (*n*)	22	18	40
Stature (cm)	187.28 [6.83]	182.83 [6.05]	185.27 [6.79]
Body mass (kg)	114.68 [6.25]	95.28 [9.61]	105.95 [12.53]
Age (years)	23.56 [3.60]	26.09 [5.32]	24.70 [4.58]
Maximal heart rate (bpm)	194.41 [13.54]	195.59 [7.41]	194.94 [11.09]
Maximal speed (m·s^−1^)	8.27 [0.28]	9.00 [0.35]	8.60 [0.48]

Data are presented as mean [standard deviation]. *n*, count; cm, centimetres; kg, kilograms; bpm, beats per minutes; m·s^−1^, meters per second.

### Design

**Figure 1:**
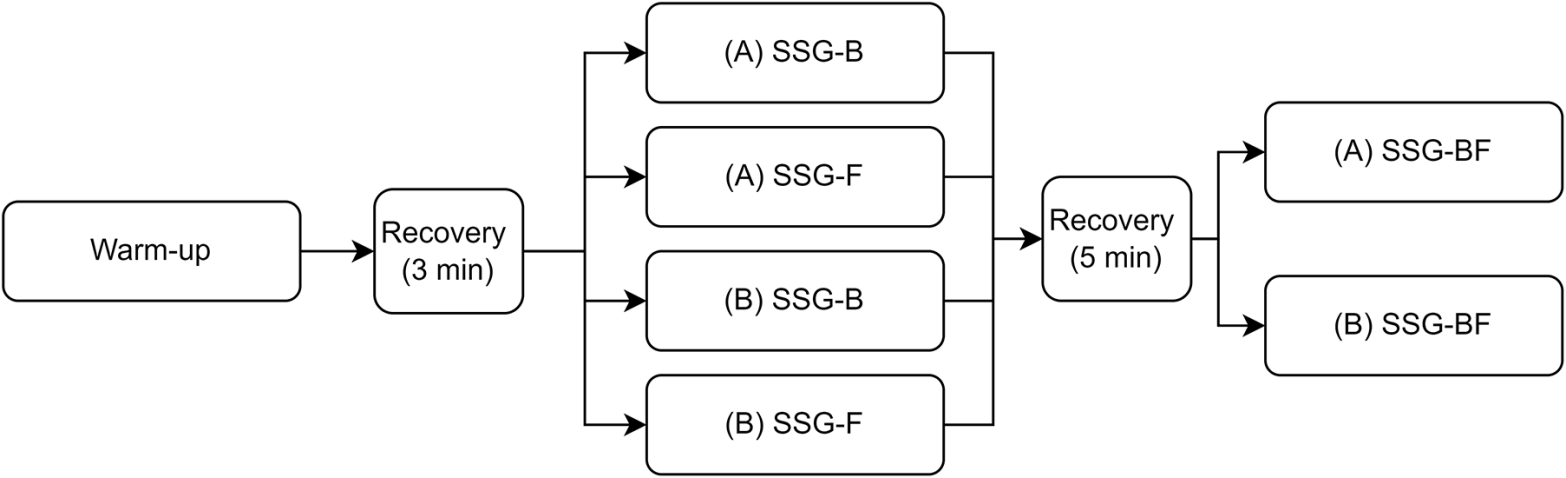
Structure of each day of data collection.

Data for this observational study were collected outdoor, on a natural grass rugby pitch, with partially sunny weather conditions, at the same time of the day, and on six different days over a 3-week period in July during the pre-season of English Gallagher Premiership 2019/2020. Each day of data collection was separated by minimum 48 h of recovery. On each day, players performed a team warm-up of approximately 12 min, consisting of light aerobic exercise (i.e., jogging), mobility covering both upper and lower body, change of direction, and both short (i.e., 5–10 m) and long (i.e., 20 m) sprint efforts, followed by two SSG formats: one for either forwards (SSG-F) or backs (SSG-B), which occurred concurrently, and one for both forwards and backs (SSG-BF) (Figure 1). Throughout the 2 weeks before the start of data collection, individuals’ maximal heart rate values were collected as the highest 5-sec average heart rate achieved during multiple training methods (i.e., 30–15 Intermittent Fitness Test, running conditioning or team rugby training sessions) (10) whilst individuals’ maximal speed was collected using a 40-m straight line sprint (11).

The SSGs were designed through the collaboration between sport scientists and elite rugby union coaches, following the constraints-led approach for practice design (12). Three SSGs were designed: one for forwards (SSG-F), one for backs (SSG-B), and one for backs and forwards (SSG-BF). The specific constraints applied to each SSG are reported in Table 2. Similar to official games, the aim of all the SSGs was for the attacking team to score a try and for the defending team to stop the opposition from scoring a try. The objectives of the SSG-B and the SSG-F were: (1) the reinforcement of specific principles and shapes of attack, and (2) the development of consistency of execution under fatigue. The objectives of the SSG-BF were: (1) the delivery of SSG that would offer repeated game-based scenarios and develop decision-making, and (2) the reinforcement of specific principles of attack. The difference between SSG-B and SSG-F was represented by the attacking shape generated by players as part of the attacking strategy. In the SSG-B, the attacking shape was similar to a straight line, whilst in the SSG-F, the attacking shape was similar to a triangle.

**Table 2:** Constraints of the small-sided games.

SSG	Task constraints						Env constraints
	Playing rules	Encou	N of players	Pitch dim	N of W and R int	W:R ratio	Playing conditions
SSG-F	- “on-side” rule: players can pass the ball exclusively backwards to players in an “on-side” position, behind an imaginary line passing through the ball and parallel to the try line. - When the *ball carrier* was touched by a *defender*, a ruck was created. The *ball carrier* had to go to the ground with a focus on a long presentation of the ball to his teammates. - One *attacking player* had to join the ruck in a low and strong position on top of the tackled player (i.e., *ball carrier*), and a *defender* had to test the *attacking player* by pushing and pulling him, thus mimicking an opponent's action of contesting for ball possession. - A turnover would occur when the *attacking player* was not low and strong under the pushing/pulling action of a *defender*, after a try was scored, when there was a poor ball delivery or when the ball was dropped.	Encouragement and feedback during rest periods	6v3 forwards	17.5 m × 15 m (length × width) RPA: 29.2 m^2^·pl^−1^	W: 2.5 min R: 75 s N of W intervals: 5	2:1	Time: 1.30 pm–3 pm; natural grass; outdoor; temperature: 17°C–20°C; partially sunny; July 2019
SSG-B	- “on-side” rule: players can pass the ball exclusively backwards to players in an “on-side” position, behind an imaginary line passing through the ball and parallel to the try line. - When the *ball carrier* was touched by a *defender*, a ruck was created. The *ball carrier* had to go to the ground with a focus on a long presentation of the ball to his teammates. - One *attacking player* had to quickly pass the ball to his teammates who would be placed wide and deep on the side of the ruck to create opportunities to attack the edges of the pitch and gain meters towards the try line. - A turnover would occur when the first receiver was not set to receive the first pass from the ruck, after a try was scored, when there was a poor ball delivery and when the ball was dropped.	Encouragement and feedback during rest periods	6v3 backs	17.5 m × 15 m (length × width) RPA: 29.2 m^2^·pl^−1^	W: 2.5 min R: 75 s N of W intervals: 5	2:1	Time: 1.30 pm–3 pm; natural grass; outdoor; temperature: 17°C–20°C; partially sunny; July 2019
SSG-BF	- “on-side” rule: players can pass the ball exclusively backwards to players in an “on-side” position, behind an imaginary line passing through the ball and parallel to the try line. - When the *ball carrier* was touched by a *defender*, a ruck was created. The *ball carrier* had to go to the ground with a focus on a long presentation of the ball to his teammates. - Two *attacking players* had to join the ruck in a low and strong position on top of the tackled player (i.e., *ball carrier*), and a *defender* had to test the *attacking players* by pushing and pulling them, thus mimicking an opponent's action of contesting for ball possession. - *Forwards* had to implement the attacking strategy practiced in SSG-F. Similarly, *backs* had to implement the attacking strategy practiced in SSG-B to create a second line of attack and find space to break the defensive line close to the edge of the pitch. - A turnover would occur after a try was scored, when there was a poor ball delivery, or the ball was dropped.	Encouragement and feedback during rest periods	11v8 backs and forwards 11 = 6 forwards, 5 backs. 8 = 4 forwards, 4 backs.	35 m × 30 m (length × width) RPA: 55.3 m^2^·pl^−1^	W: 3.5 min R: 90 s N of W intervals: 3	2.3:1	Time: 1.30 pm–3 pm; natural grass; outdoor; temperature: 17°C–20°C; partially sunny; July 2019

SSG, small-sided game; F, forwards; B, backs; Encou, encouragement; N, number; W, work; R, rest; Env, environmental; RPA, relative playing area.

### Methodology

Internal load was assessed using chest strap heart rate monitors (Polar H1, Polar, Kempele, Finland) (4) with Stagno's training impulse (TRIMP) as outcome measure (13) (Table 3). Stagno's TRIMP is determined using individual's maximal heart rate, and pre-determined heart rate zones and weighting factors (13). Stagno's TRIMP demonstrated a correlation with changes in maximal oxygen uptake (*r* = 0.80, *p* = 0.017) and velocity at onset of blood lactate accumulation (*r* = 0.71, *p* = 0.024) over a period of training, thus suggesting its validity to monitor internal load (13). Additionally, the weighting factors for Stagno's TRIMP were originally derived from laboratory testing, thus representing a more robust measure in comparison with Edward's or Lucia's TRIMP which instead use arbitrary weighting factors (13). Furthermore, Stagno's TRIMP has been previously used in field-based team sports (e.g., rugby union, soccer) (13, 17, 18).

**Table 3:** Description of the internal and external load parameters collected.

Outcome measures		
Load	Parameter	Description
Internal load	Stagno's TRIMP (AU)	Training impulse is determined by multiplying the time spent in pre-determined heart rate zones, based on a percentage of individual's maximal heart rate, by a weighting factor and then adding together the resulting values (13). <continuous variable>
External load	Total distance (m)	Total distance covered in meters. <continuous variable>
	HSR (>61%) distance (m)	Distance covered in meters above 61% of an individual's maximal speed. <continuous variable>
	Avg accel decel (m·s^−2^)	Average acceleration deceleration is determined by averaging the absolute values of all the accelerations and deceleration detected by the GPS over a period of time (14). <continuous variable>
	PlayerLoad™ (AU)	PlayerLoad™ is a vector magnitude derived from the square root of the sum of the squared instantaneous rates of change in acceleration in each of the three axes of movement (i.e., x, y, z) (15). <continuous variable>
	PlayerLoad™ slow (<2 m·s^−1^) (AU)	PlayerLoad™ slow is the PlayerLoad™ accumulated during activities performed at a speed lower than 2 m·s^−1^ (16). <continuous variable>
	Get-up (*n*)	Get-up represents the number of times a player is getting up from the ground, thus transitioning from a lying to a standing position. <discrete variable>
	First-man-to-ruck (*n*)	First-man-to-ruck represents the number of times an attacking player is going on top of the tackled players in a crouch position to prevent a defender from stealing the ball. Whilst in a crouch position, the attacking player is pushed and pulled by a defender player and his goal is to resist to this external perturbation, thus maintaining his position. <discrete variable>

HSR, high-speed running; AU, arbitrary units; Avg, average; accel, acceleration; decel, deceleration; TRIMP, training impulse; *n*, number of.

External load was quantified using GNSS 10 Hz and tri-axial accelerometer 100 Hz (Vector S7, Catapult Sports, Catapult Innovations, Melbourne, Australia), and video camera 25 Hz (Sony NXCAM Avchd MPEG2 SD, Sony, Tokyo, Japan). Total distance covered (m), high-speed (>61%) running distance (m), average acceleration-deceleration (m·s^−2^) (Table 3) were collected using GNSS 10 Hz, processed in OpenField console (v3.3.0), and exported from OpenField cloud (Catapult Sports, Catapult Innovations, Melbourne, Australia). The validity and reliability of GNSS 10 Hz devices have been extensively investigated (14, 19, 20). Devices were turned on outside 15 min before the start of the session to optimise satellite signal. On players’ arrival, the devices were placed in a custom vest produced by the GNSS manufacturer, and each player was assigned a unique GNSS device throughout the duration of the study to control for inter-device reliability. The mean and standard deviation (SD) for number of satellites and horizontal dilution of precision were mean = 12.5, SD = 1.46, and mean = 0.67, SD = 0.06, respectively.

Tri-axial accelerometers were embedded within the GNSS devices and used to collect PlayerLoad™ (AU) and PlayerLoad™ Slow (<2 m·s^−1^) (AU) (Table 3). PlayerLoad™ has shown a good test-retest reliability when recorded at the scapulae (ICC: 0.93, CV: 5.9%) (15). Additionally, both PlayerLoad™ and PlayerLoad™ Slow (<2 m·s^−1^) differentiated among playing positions in Australian football players, and PlayerLoad™ Slow (<2 m·s^−1^) correlated with the number of collisions in under-18 rugby union forwards (*r* = 0.70) and backs (*r* = 0.61) during official games, thus supporting the validity of these measures to quantify external load (7, 16).

A video camera was utilised to collect get-up (count) and first-man-to-ruck (count) (Table 3). Videos from the training sessions were imported into Catapult Vision (Catapult Sports, Catapult Innovations, Melbourne, Australia) where the single events were visually coded by the first author (MZ). Intrarater reliability was assessed by re-coding a single day of data collection chosen at random after a 6-week washout period. A two-way agreement mixed-effects model was used to determine intraclass correlation coefficients (ICC) for both get-up and first-man-to-ruck (21), using the icc() function in the *irr* package in R v4.0.3 (22). Intraclass correlation was “excellent” for both get-up and first-man-to-ruck with an ICC [95%CI] of 0.98 [0.98–0.99] and 0.99 [0.98–0.99], respectively (23).

### Statistical analysis

Analysis was conducted using statistical computing language R v4.0.3 (22) within RStudio (RStudio Team, 2018, v1.2.1335). Before any formal statistical analysis, data were investigated to detect potential outliers, which were defined as “*an observation (or subset of observations) which appear to be inconsistent with the remainder of that set of data”* (24, p. 4). This process was conducted due to initial concerns about the possible detachment or misplacement of the heart rate sensor due to the motion of getting down to and up from the ground, which could result in extremely low or zero values recorded. The potential outlier identification process followed the guidelines reported by Aguinis et al. (25), using both univariate (i.e., box plots, three standard deviations, modified *z*-scores) and multivariate techniques (i.e., leverage, studentised deleted residuals, changes in model fit using coefficient of determination *R*^2^ and Akaike Information Criteria, changes in model predictors using DFBETAS, DFFITS, Cook's distance). In addition, assuming that the heart rate sensor was appropriately connected and that a player spent the total duration of the SSG bout within 65% and 71% of his maximal heart rate (i.e., moderate exercise) which is associated to a weighting factor of 1.25, the lowest Stagno's TRIMP values could have been 187.50 AU for SSG-B and SSG-F (i.e., 187.50 AU = 150 s, the duration of each SSG-B and SSG-F, multiplied by 1.25 weighting factor) and 262.50 AU for SSG-BF (i.e., 262.50 AU = 210 s × 1.25) (13). These values were also used as reference for outlier identification.

Due to the longitudinal design of the study with within-subjects repeated measures, general linear mixed-effects models were used to investigate the data using *lme4* and *stats* packages (22, 26). The initial models were designed including all the external load variables as independent variables (fixed effects), the internal load variable as dependent variable, and the intercept as random effect, which was allowed to vary based on date of data collection, SSG bout number, and players involved (27, 28). The likelihood-based approach was utilised for model building and statistical inference (29, 30). Model building was driven by consideration of various aspects, with a primary role played by simplicity of the model, Akaike Information Criteria, likelihood ratio test, and visual assessment of the regularity of the log-likelihood function (29). These strategies were also implemented to resolve possible multicollinearity among variables using model comparison (31). Multicollinearity was identified when the variance inflation factor for an independent variable showed a value higher than 2 (31). Model inference was based on the log-likelihood function utilising the maximum likelihood estimate (MLE), which represents the point of global maximum of the function, and its standard error (SE), which is derived from the squared root of the observed Fisher information (i.e., the negative of the second derivative of the function at the MLE point) (29, Section 3.7). In addition, the log-likelihood function was used to determine the profile likelihood confidence intervals at 95% level (95%PLCI) to identify a range of parameter values compatible with the data under the specified model (29, Section 4.5). Profile likelihood confidence intervals are derived from the distribution of likelihood ratio tests [i.e., 2(max log-likelihood—log-likelihood)] which can be approximated to a probability distribution (e.g., chi-squared), and the 95% level is the selected quantile of that probability distribution (29, Section 4.5). Maximum likelihood estimates and SE can also be used to test the hypothesis that a model coefficient differs from zero by calculating Wald statistics (i.e., *t* values) (29, Section 4.4). A Wald statistic close to zero suggests that the data is consistent with the hypothesis of the coefficient being zero, whereas more extreme values suggest evidence against the hypothesis of the coefficient being zero (32). The lack of effect of a model parameter was identified by a Wald statistic close to zero and a 95%PLCI including zero.

## Results

The process of potential outlier detection led to identify as outliers 18 observations in the SSG-B, 7 observations in the SSG-F, and 19 observations in the SSG-BF data frames. These observations were considered to be produced by a misplaced heart rate sensor. Therefore, as they were generated by measurement error, they were removed from the data frames, thus resulting in a sample size of 437 observations for SSG-B, 535 observations for SSG-F, and 640 observations for SSG-BF. The descriptive statistics for the characteristics of each SSG design are reported in Table 4.

**Table 4:** Descriptive statistics (mean [standard deviation]) for the internal and external load characteristics for the three small-sided game designs.

Variable	SSG-B	SSG-F	SSG-BF
TRIMP (AU)	521.74 [111.89]	572.86 [103.82]	641.75 [153.18]
Total distance (m)	274.53 [25.39]	231.21 [23.35]	308.57 [50.50]
HSR distance (m)	2.04 [4.26]	0.89 [2.23]	3.76 [6.94]
Avg acc dec (m·s^−2^)	0.74 [0.07]	0.67 [0.06]	0.60 [0.07]
PlayerLoad™ (AU)	29.40 [3.85]	27.56 [3.59]	31.43 [5.98]
PlayerLoad™ Slow (AU)	11.01 [1.99]	13.55 [2.14]	11.83 [2.28]
Get-up (count)	1.46 [1.87]	1.32 [1.69]	0.73 [1.21]
First-man-to-ruck (count)	-	1.16 [1.62]	0.78 [1.30]

Data are presented as mean and [standard deviation].

**Figure 2:**
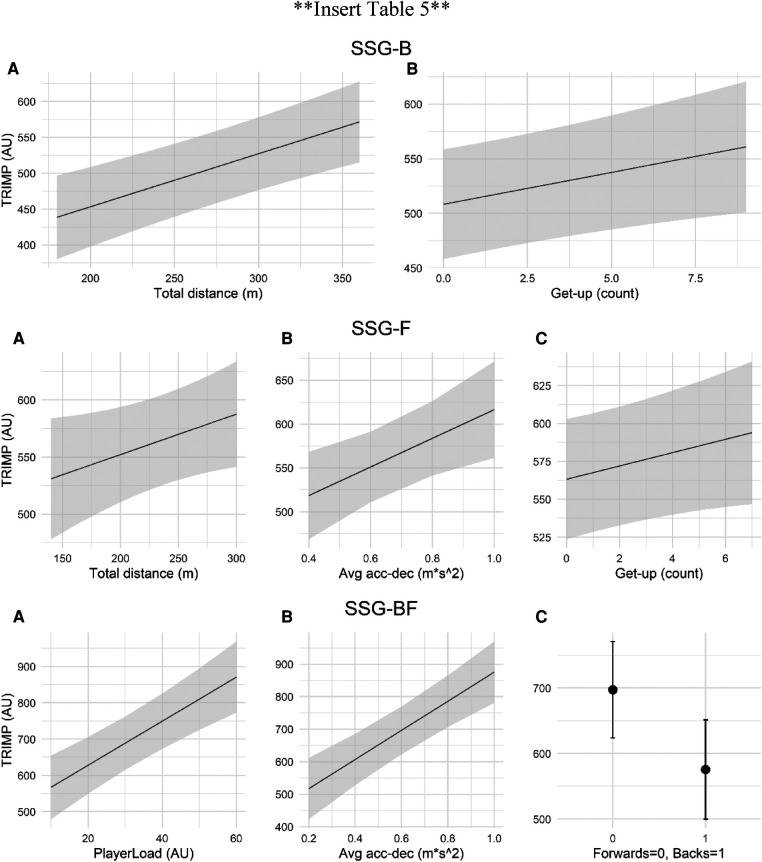
Representation of the model estimates for each fixed-effect (**A-C**) in the three small-sided game designs (i.e., SSG-B, SSG-F, SSG-BF).

The process of model selection led to the final models which are reported with and without outliers in the Supplementary Material. Models without error outliers were used for inference and their fixed effects are presented in Table 5. The model selection process showed that two external load variables (i.e., total distance, get up) were associated with internal load during SSG-B, three external load variables (i.e., average acceleration deceleration, total distance, get up) were associated to internal load in SSG-F, and two external load variables (i.e., average acceleration deceleration, PlayerLoad™) plus playing position (i.e., forward, back) were associated with internal load in SSG-BF (Table 5). The fixed effects of each model can be interpreted as traditional general linear models, where the MLE represents the change in Stagno's TRIMP for a one unit increase in the fixed effect, with the intercept being the average Stagno's TRIMP (27). For instance, in SSG-B, TRIMP increases by 5.84 AU for every get-up performed, whereas it increases by 0.74 AU for every meter covered during each bout (Table 5). A visual representation of the model estimates is provided in Figure 2.

**Table 5:** Fixed effects of the models without error outliers for the three small-sided game designs.

Models fixed effects without error outliers					
Drill	Fixed effects	MLE	SE	*t*	95%PLCI
SSG backs (SSG-B)	Intercept	305.50	50.26	6.08	[205.46, 405.51]
	Total distance	0.74	0.16	4.72	[0.43, 1.05]
	Get-up	5.84	2.26	2.58	[1.41, 10.37]
SSG forwards (SSG-F)	Intercept	371.42	55.68	6.67	[261.85, 481.51]
	Total distance	0.35	0.19	1.88	[−0.02, 0.73]
	Avg acc dec	163.17	58.15	2.80	[48.33, 276.62]
	Get-up	4.38	2.39	1.83	[−0.29, 9.08]
SSG backs & forwards (SSG-BF)	Intercept	236.88	50.60	4.68	[137.36, 337.68]
	Avg acc dec	447.27	75.25	5.94	[297.27, 595.93]
	PlayerLoad™	6.08	1.15	5.28	[3.78, 8.38]
	Forward0 back1	−121.94	29.03	−4.20	[−179.54, −64.74]

MLE, maximum likelihood estimate; SE, standard error; PLCI, profile likelihood confidence intervals; *t*, Wald statistic; Forward0 back1, forwards were coded as 0; backs were coded as 1; Avg acc dec, average acceleration deceleration.

## Discussion

The current study aimed to investigate which external load variables influenced internal load, and the relation between internal load and its associated external load variables during three different rugby union SSGs. The present findings showed that the design of the SSG influenced which external load variables were associated with internal load. Furthermore, in the SSG-BF, internal load differed between backs and forwards. Therefore, in these specific SSGs, practitioners should manipulate different constraints to elicit a certain physiological response in their players and playing position should also be taken into account.

Previous research in rugby league, association football, and Australian rules football, investigated the association between internal and external load variables by aggregating data from multiple training methods (e.g., technical, tactical, SSG training) (3, 5, 6). However, these findings may not be applicable when practitioners aim to use external load variables to design specific drills as different training methods may be characterised by different internal external load associations. This is supported by the findings of the present study where the external load variables associated with internal load changed based on the specific design of the SSG investigated (Table 5). Therefore, the findings derived from the aggregation of multiple training methods may not be specific enough to allow practitioners to manipulate constraints in order to target specific external load variables, and elicit the desired physiological response during specific training drills, for instance SSG (3).

In the SSG-B, internal load was associated with total distance covered and number of get-ups (Table 5). Internal load (i.e., TRIMP) increased by 0.74 AU for each one-meter increase in total distance covered, thus increasing total distance would result in higher internal load (Table 5). In addition, an increase in the number of get-ups by one would lead to an increase of 5.84 AU in Stagno's TRIMP, thus showing a stronger effect on internal load in comparison with total distance covered (Table 5). In the SSG-F, internal load was associated with total distance covered, number of get-ups, and average acceleration-deceleration (Table 5). Similarly to SSG-B, internal load increased by 0.35 AU for every one-meter increase in total distance, and by 4.38 AU for every one-unit increase in the number of get-ups (Table 5). As values of average acceleration-deceleration may range between 0.1 and 0.9, an increase of 0.1 m·s^−2^ in average acceleration-deceleration would lead to a 16.32 AU increase in TRIMP (i.e., 16.32 AU = 163.17 × 0.1 m·s^−2^) (Table 5). Therefore, during SSG-F, average acceleration-deceleration showed the strongest effect on TRIMP.

The substantial contribution of number of get-ups to internal load may be due to the involvement of upper limbs in the motion of getting up from the ground (33). Irrespectively of exercise intensity and oxygen uptake, heart rate has been shown to be 20% higher during upper-body exercises in comparison with lower-body exercises (33). In addition, the rapid transition from a lying to a standing position may reduce the blood volume in the left ventricle, thus reducing stroke volume, and ultimately increasing heart rate (34). Furthermore, this finding emphasises the importance of quantifying actions that may not be measurable by typical external load methods, such as GNSS and tri-axial accelerometers.

In the SSG-BF, internal load was associated with PlayerLoad™, average acceleration-deceleration, and playing position (i.e., back, forward) (Table 4). Internal load showed an increase of 44.73 AU for an increase of 0.1 m·s^−2^ in average acceleration-deceleration (i.e., 44.73 AU = 447.27 × 0.1 m·s^−2^) whereas a one-AU increase in PlayerLoad™ resulted in a 6.08 AU increase in TRIMP (Table 5). In addition, backs were characterised by TRIMP values of 121.94 AU lower in comparison with forwards (Table 5). The contribution of accelerations and decelerations, total distance covered, and PlayerLoad™ on internal load is supported by previous research in association football and rugby league (5, 8). The different internal load between backs and forwards may be due to the design of the SSG-BF and its objective of offering repeated game-based scenarios. Previous research has shown that during an official rugby union game, forwards spent substantially more time at 85%–95% of their maximal heart rate in comparison with backs (*p* < 0.05) (1). Consequently, the SSG-BF may have been able to reproduce the characteristics of official games, thus leading to a different internal load in backs and forwards.

In terms of methodology, most of previous research identified the issue of multicollinearity as a source of biased model coefficients, and dealt with it accordingly, for instance *via* principal component analysis (3, 5). However, an additional issue when using linear models to determine the association between dependent and independent variables is the influence of error outliers (i.e., outliers generated by measurement errors) on model coefficients, thus resulting in biased estimates (25). Findings from the current study showed that the inclusion of error outliers led to different MLE for the fixed effects, with systematically larger SE (Supplementary Material). Furthermore, the model for SSG-B with the inclusion of error outliers resulted in a singularity issue. Singularity refers to the scenario where one of the linear mixed-effects model parameters is approximately or indeed zero, thus suggesting potential overfitting in terms of random effects and resulting in inaccurate PLCI, likelihood ratio tests, and Wald statistics (35). Therefore, practitioners should also assess the presence of outliers in their data in addition to multicollinearity to enhance the accuracy of their inferences.

## Practical applications

Based on the SSG investigated in the current study, practitioners may need to manipulate different constraints to elicit a certain cardiovascular response based on the specific SSG design. Constraints aimed to increase the number of get-ups and average acceleration deceleration may enhance the internal load characteristics of the SSG. Furthermore, practitioners should be aware that the SSG-BF specifically designed in this study may offer a different internal load to backs and forwards. A limitation of the current study was the exclusive use of Stagno's TRIMP to measure internal load. No other internal load measures (e.g., session-RPE) could be collected as rugby union coaches did not want to disrupt the flow of the session. Furthermore, as Stagno's TRIMP implements the same heart rate zones for all subjects, it may not be specific enough to represent the blood lactate-heart rate profile of each individual. Finally, it is recommended to investigate the presence of potential outliers in heart rate data to make more accurate inferences.

## Conclusion

The current study showed that the association between internal and external load variables changed based on the specific design of the SSG in professional rugby union players. In the specific SSG-B investigated, heart rate was associated with total distance covered and number of get-ups. In the specific SSG-F investigated, heart rate was associated with average acceleration-deceleration, total distance covered, and number of get-ups. In the specific SSG-BF investigated, heart rate was associated with average acceleration-deceleration, PlayerLoad™, and playing position. Furthermore, in the SSG-BF, forwards and backs showed a different internal load. These findings may support sports scientist and rugby union coaches in the process of SSG design.

## Data Availability

The datasets presented in this study can be found in online repositories. The names of the repository/repositories and accession number(s) can be found below: https://github.com/marcozan93/Research-data/tree/main/study_3.
