# Supplementary material for: The contributing external load factors to internal load during small-sided games in professional rugby union players
Source: Front Sports Act Living. 2023 Feb 15;5:1092186. doi: 10.3389/fspor.2023.1092186 (PMC9975384; doi:10.3389/fspor.2023.1092186)
Supplement: Supplementary file 1 [file Table1.docx]

SUPPLEMENTAL MATERIAL

Model summary for the three small-sided games with and without outliers.

| **Drill** | **Model parameters without error outliers** | | | | | **Model parameter with error outliers** | | | |
| --- | --- | --- | --- | --- | --- | --- | --- | --- | --- |
| SSG backs (SSG-B) | **Fixed effects** | **MLE** | **SE** | **t** | **95%PLCI** | **MLE** | **SE** | **t** | **95%PLCI** |
|  | Intercept | 305.50 | 50.26 | 6.08 | [205.46, 405.51] | 333.62 | 65.51 | 5.09 | [204.93, 464.65] |
|  | Total distance | 0.74 | 0.16 | 4.72 | [0.43, 1.05] | 0.58 | 0.21 | 2.73 | [0.15, 0.99] |
|  | Get-up | 5.84 | 2.26 | 2.58 | [1.41, 10.37] | 6.84 | 3.31 | 2.06 | [0.36, 13.38] |
|  | **Random effects** | **SD** | | **95%PLCI** | | **SD** | | **95%PLCI** | |
|  | Date (int) | 36.72 | | [15.74, 77.55] | | 40.96 | | [13.82, 88.91] | |
|  | SSGB (int) | 16.53 | | [0.00, 43.21] | | 21.24 | | [5.77, 50.93] | |
|  | Player (int) | 76.31 | | [53.41, 111.03] | | 85.81 | | [58.40, 126.38] | |
|  | Date:SSGB (int) | 23.31 | | [14.36, 36.55] | | 20.58 | | [6.54, 36.12] | |
|  | Date:player (int) | 34.01 | | [25.52, 43.69] | | 60.81 | | [48.03, 76.21] | |
|  | SSGB:player (int) | 26.55 | | [14.44, 38.00] | | 0.00 | | [0.00, 18.67] | |
|  | Residuals | 53.09 | | [48.25, 58.43] | | 78.52 | | [72.76, 84.70] | |
| SSG forwards (SSG-F) | **Fixed effects** | **MLE** | **SE** | **t** | **95%PLCI** | **MLE** | **SE** | **t** | **95%PLCI** |
|  | Intercept | 371.42 | 55.68 | 6.67 | [261.85, 481.51] | 328.40 | 66.38 | 4.95 | [197.56, 460.32] |
|  | Total distance | 0.35 | 0.19 | 1.88 | [-0.02, 0.73] | 0.54 | 0.23 | 2.36 | [0.08, 0.99] |
|  | Avg acc dec | 163.17 | 58.15 | 2.80 | [48.33, 276.62] | 150.38 | 71.44 | 2.10 | [9.33, 289.68] |
|  | Get-up | 4.38 | 2.39 | 1.83 | [-0.29, 9.08] | 7.24 | 2.88 | 2.51 | [1.61, 12.89] |
|  | **Random effects** | **SD** | | **95%PLCI** | | **SD** | | **95%PLCI** | |
|  | Date (int) | 31.18 | | [8.79, 67.26] | | 28.74 | | [0.00, 65.12] | |
|  | Player (int) | 62.76 | | [44.18, 88.84] | | 67.33 | | [46.56, 95.77] | |
|  | Date:SSGB (int) | 27.81 | | [20.01, 39.74] | | 28.97 | | [20.25, 41.99] | |
|  | Date:player (int) | 53.16 | | [44.51, 63.73] | | 61.25 | | [51.05, 73.63] | |
|  | Residuals | 49.18 | | [45.81, 52.64] | | 61.45 | | [57.28, 65.74] | |
| SSG backs & forwards (SSG-BF) | **Fixed effects** | **MLE** | **SE** | **t** | **95%PLCI** | **MLE** | **SE** | **t** | **95%PLCI** |
|  | Intercept | 236.88 | 50.60 | 4.68 | [137.36, 337.68] | 226.55 | 51.86 | 4.37 | [124.89, 328.50] |
|  | Avg acc dec | 447.27 | 75.25 | 5.94 | [297.27, 595.93] | 371.51 | 88.95 | 4.18 | [193.01, 545.82] |
|  | PlayerLoad^TM^ | 6.08 | 1.15 | 5.28 | [3.78, 8.38] | 7.16 | 1.33 | 5.37 | [4.53, 9.84] |
|  | Forward0 back1 | -121.94 | 29.03 | -4.20 | [-179.54,  -64.74] | -111.07 | 31.17 | -3.56 | [-172.77,  -49.72] |
|  | **Random effects** | **SD** | | **95%PLCI** | | **SD** | | **95%PLCI** | |
|  | Date (int) | 66.83 | | [19.59, 135.68] | | 53.06 | | [0.00, 114.39] | |
|  | SSGB (int) | 24.43 | | [0.00, 87.92] | | 17.97 | | [0.00, 72.05] | |
|  | Player (int) | 83.87 | | [62.84, 108.71] | | 80.41 | | [53.86, 108.85] | |
|  | Date:SSGB (int) | 47.54 | | [31.09, 81.59] | | 45.54 | | [29.57, 76.74] | |
|  | Date:player (int) | 69.59 | | [59.96, 80.67] | | 117.15 | | [103.50, 133.25] | |
|  | Residuals | 63.97 | | [59.71, 68.49] | | 75.85 | | [70.09, 81.06] | |
| Notes: MLE: maximum likelihood estimate; SE: standard error; SD: standard deviation; PLCI: profile likelihood confidence intervals; t: Wald statistic; Forward0 back1: forwards were coded as 0, backs were coded as 1; SSGB: small-sided game bout; Avg acc dec: average acceleration-deceleration; A (int): random effect of A relative to the intercept; A:B (int): random effect of B within A relative to the intercept. | | | | | | | | | |
